# Effect of timing of school enrollment on physical fitness in third graders

**DOI:** 10.1038/s41598-022-11710-x

**Published:** 2022-05-12

**Authors:** Thea Fühner, Urs Granacher, Kathleen Golle, Reinhold Kliegl

**Affiliations:** Division of Training and Movement Sciences, Faculty of Human Sciences, University of Potsdam, Am Neuen Palais 10, Building 12, 14469 Potsdam, Germany

**Keywords:** Physiology, Health care

## Abstract

Timing of initial school enrollment may vary considerably for various reasons such as early or delayed enrollment, skipped or repeated school classes. Accordingly, the age range within school grades includes older-(OTK) and younger-than-keyage (YTK) children. Hardly any information is available on the impact of timing of school enrollment on physical fitness. There is evidence from a related research topic showing large differences in academic performance between OTK and YTK children versus keyage children. Thus, the aim of this study was to compare physical fitness of OTK (N = 26,540) and YTK (N = 2586) children versus keyage children (N = 108,295) in a representative sample of German third graders. Physical fitness tests comprised cardiorespiratory endurance, coordination, speed, lower, and upper limbs muscle power. Predictions of physical fitness performance for YTK and OTK children were estimated using data from keyage children by taking age, sex, school, and assessment year into account. Data were annually recorded between 2011 and 2019. The difference between observed and predicted z-scores yielded a *delta* z-score that was used as a dependent variable in the linear mixed models. Findings indicate that OTK children showed poorer performance compared to keyage children, especially in coordination, and that YTK children outperformed keyage children, especially in coordination. Teachers should be aware that OTK children show poorer physical fitness performance compared to keyage children.

## Introduction

The importance of physical fitness for children’s health is undisputed^1^. According to Caspersen et al.^2^, physical fitness can be categorized as health- (e.g., cardiorespiratory endurance, muscular endurance, muscular strength, body composition, and flexibility) or skill-related fitness (e.g., agility, balance, coordination, speed, [muscle] power, and reaction time). There is evidence from original research^3^, systematic reviews, and meta-analyses^4,5^ that cardiorespiratory endurance and muscular strength are positively associated with markers of physical health (e.g., body mass index, waist circumference, skinfold thickness, cardiovascular disease risk score) in youth. Accordingly, it is important to regularly monitor and evaluate children’s physical fitness to identify potential deficits in physical fitness as early as possible. Recent studies on global secular trends in youth physical fitness indicated physical fitness declines particularly for measures of cardiorespiratory endurance. This trend additionally emphasizes the relevance of physical fitness testing^6,7^.

Physical fitness tests represent an easy-to-administer, reliable, and valid means to assess and evaluate children’s physical fitness in large scale studies conducted in sport clubs or schools^8^. Several studies from around the globe^8–17^ showed developmental increases in physical fitness from childhood to adolescence^8,10–12,15,17^. Irrespective of age, boys outperform girls in most components of physical fitness^9–17^, except for balance^10,17^ and flexibility^9–15,17^.

The available studies on physical fitness development have been conducted in youth aged 5–18 years. In these studies, children and adolescents were matched into 1-year age groups. This age grouping system is also evident in many settings of children’s everyday life. For instance, children are matched in 1-year age teams within sport clubs or in grades within schools. However, this age grouping system is not without limitations because of differences in relative age depending on the specific cut-off date under consideration. For schools in general, the cut-off date of initial school enrollment is specific to the country under investigation. For instance, in the Federal State of Brandenburg, Germany the official and initial school enrollment date is September 30th. Accordingly, children are enrolled to school (i.e., first grade) if they are aged between 6 years and 0 months and 6 years and 11 months on September 30th of the respective year (i.e., keyage children in first grade). Thus, children who are born on September 30th or slightly later are at the extreme end, i.e., almost 1 year older than their classmates who are born in August. These differences in the birthdate may have an impact on anthropometrics (e.g., body height, body mass) and physical fitness (e.g., muscular strength, power, cardiorespiratory endurance, or speed)^18^ because physical fitness performance increases with age from childhood to adolescence^8,10–12,15,17^. Thus, within a 1-year age-group, the relatively older children (i.e., born near the cut-off date) may outperform their relative younger classmates (i.e., born later to the cut-off date) because of their relatively older age^18,19^. In fact, a previous study conducted with keyage third graders (i.e., children aged 8 years and 0 months to 8 years and 11 months) has shown that physical fitness increased linearly with chronological age^20^. Furthermore, even within the single ninth year of life, the relatively older children (i.e., aged 8 years and 6 months to 8 years and 11 months) significantly outperformed the younger children (i.e., aged 8 years and 0 months to 8 years and 5 months) in physical fitness^20^.

Within one school-grade, there are keyage children as well as younger- (YTK) or older-than-keyage (OTK) children. This is due to early or late school enrollment, skipping or repetition of a school year. With reference to our data, age ranged from 5 years and 11 months to 14 years and 5 months for our study sample that included YTK and OTK children. Given that there are already large differences in physical fitness within the group of keyage children^20^, the question arises as to physical fitness performance of YTK and OTK children. To the authors’ knowledge there is hardly any information available in the literature on differences in physical fitness of YTK and OTK children versus keyage children. A major goal of physical education is to create a learning setting for each child according to his/her individual needs to ensure a holistic development. Thus, findings on physical fitness performance of YTK and OTK children provide valuable information to promote physical fitness according to the child´s individual needs. For instance, children who show delayed physical fitness development should receive additional health and fitness programs to compensate their deficits in physical fitness. Furthermore, given that grading systems are only available for keyage children, findings of this study can be used to individually grade physical fitness according to age, sex, and timing of school enrollment.

Information from a related research topic shows large differences in academic performance between OTK and YTK children versus keyage children^21–23^. For instance, in a study including 1144 German primary school children, Urschitz et al.^23^ reported that especially OTK children aged > 9 years compared with keyage children showed poor academic performance in terms of grades in mathematics, science, reading, spelling, and handwriting. In a study including 3,684 Australian high school students aged 14 years, Martin^22^ reported that YTK children scored significantly better in academic performance (i.e., performance in literacy and numeracy) than keyage children. However, as already mentioned this has not yet been examined for physical fitness. Therefore, the aim of this cross-sectional study was to compare physical fitness of OTK and YTK children versus keyage children in a sample of German primary school children taking age, sex, school, and assessment year into account. With reference to the relevant school-based studies on differences in academic performance of OTK and YTK children versus keyage children^21–23^, we hypothesized that OTK children show poorer and YTK children better physical fitness performance compared with keyage children.

## Methods

### Experimental approach

This cross-sectional study is part of the ongoing EMOTIKON research project (www.uni-potsdam.de/en/emotikon). Physical fitness tests were conducted every year between September and November starting in 2011. Physical fitness tests were also administered in 2009 and 2010, but later in the school year that is between March and April. Due to the seasonal variation in physical fitness these data were not included.

### Population

Since 2009, all third graders living in the Federal State of Brandenburg, Germany were tested annually for their physical fitness. This cross-sectional study was mandated and approved by the Ministry of Education, Youth and Sport of the Federal State of Brandenburg, Germany. The Brandenburg School Law requires that parents are comprehensively informed prior to the start of the study. Consent is not needed given that the tests are obligatory for both, children and schools^24^. None of the authors included in the author list had access to personally identifiable information on the children. The authors received the data absolutely anonymized from the Ministry of Education, Youth and Sport of the Federal State of Brandenburg, Germany. Research was conducted according to the latest Declaration of Helsinki^25^.

To compare physical fitness development of YTK and OTK children with that of keyage children, we used physical fitness data recorded between 2011and 2019.

- 2586 YTK children aged 7 years and 0 months to 7 years and 11 months
- 108,296 keyage children aged 8 years and 0 months to 8 years and 11 months
- 26,540 OTK children aged 9 years and 0 months to 9 years and 11 months

Selection into keyage, OTK, and YTK groups was strictly based on children’s birthdate relative to the legal date for school enrollment (i.e., September 30th in the Federal State of Brandenburg for all assessment years). Thus, on September 30th, keyage third graders ranged between 8 years and 0 months to 8 years and 11 months. YTK children were younger, and OTK children were older.

The selection of keyage children has been described in a previous publication of our research group^20^. Data from an earlier study were used as a reference for OTK and YTK children. Initially, 30,253 OTK children were included in the data base: 2842 were excluded due to age. The excluded third-graders ranged from 10 years and 1 month to 14 years and 5 months. Another 27 students were excluded due to adverse health events as reported by the responsible teacher (e.g., physical disability, autism spectrum). Finally, 844 students were considered outliers and outside + /− 3 SD of their group x sex x test cell. Finally, 26,540 OTK children were included in the analyses (88.7%). For YTK children, initially 2654 YTK were eligible to be included in the data base. From this initial sample, 28 were excluded due to age because they ranged from 5 years and 11 months to 6 years and 11 months. Moreover, 40 children were considered outliers and beyond + /− 3 SD in their group × sex × test cell. Finally, 2586 YTK children were included in the analyses (97.4%).

### Physical fitness tests

Physical fitness was assessed using the specific EMOTIKON test battery^20^. These tests evaluated cardiorespiratory endurance (i.e., 6-min-run test), coordination (i.e., star-run test), speed (i.e., 20-m linear sprint test), lower (powerLOW [i.e., standing long jump test]), and upper limbs muscle power (i.e., powerUP [ball-push test]). The EMOTIKON test battery officially includes six tests. In 2016, the assessment of flexibility (i.e., stand-and-reach test) was stopped and the assessment of balance (i.e., single-leg balance test with eyes closed) was included^26^. Due to the much smaller number of scores and their confound with assessment year, these two tests were not included in the analyses.

Physical fitness tests were administered by qualified physical education teachers and conducted during the regular physical education classes. All physical education teachers received standardized test instructions for the assessment (www.uni-potsdam.de/en/emotikon/projekt/methodik—for further information on the test protocols). Furthermore, all physical education teachers participated in advanced training programs about standardized physical fitness assessment. Tests were always conducted in the morning between 8 and 12 am. Prior to testing, all third-graders performed a standardized warm-up program consisting of different running exercises (e.g., side-steps) and small games (e.g., playing tag).

#### Cardiorespiratory endurance

Cardiorespiratory endurance was assessed using the 6-min-run test. Participating children had to run the furthest distance during the 6 min test time around a volleyball field (54 m) at a self-paced velocity. The test instructor provided split times every minute. After the 6 min, maximal distance covered in meters to the nearest nine meters was recorded and used as dependent variable. High test–retest reliability was reported for the 6-min-run test with an intraclass correlation coefficient (ICC) of 0.92 in children aged 7–11 years^27^.

#### Coordination

Coordination under time pressure was evaluated using the star-run test. During the star-run test, the participating children had to complete a parkour with different running techniques (i.e., running forwards, running backwards, side-steps) as quickly as possible. The star shaped parkour (9 m × 9 m) consisted of four spikes. Each spike and the center of the star were marked with a pylon. The participants started in the middle of the star. First, they had to run forward to the first pylon and backward to the middle. Next, they had to do side-steps to the second pylon on the right side and side-steps back to the middle. Then, they had to run backward to the third pylon and forward to the middle. Finally, they had to do side-steps to the fourth pylon on the left side and side-steps back to the middle. The participants had to touch each pylon within the parkour with the hand. The whole covered distance was 50.912 m. Time for test completion in seconds to the nearest 1/10 s was taken using a stopwatch and used as dependent variable in the analysis. The participants had two test trials of which the best test trial in terms of time until test completion was kept for analysis. The star run test was reliable (test–retest) for children aged 8–10 years with an ICC of 0.68^28^.

#### Speed

Speed was assessed using the 20-m linear sprint test. The participating children started from a standing position with one foot right behind the starting line. After an acoustic signal, they had to sprint as fast as possible over a distance of 20 m. Time for test completion in seconds to the nearest 1/10 s was taken using a stopwatch and used as dependent variable in the analysis. The participants had two test trials of which the best trial was taken for further analysis in terms of the time until test completion. Test–retest reliability has been reported to be high for children aged 7–11 years with an ICC of 0.90^27^.

#### Lower limbs muscle power (PowerLOW)

PowerLOW was assessed through the standing long jump test. The participating children had to jump as far as possible from a frontal position. Arm swing prior to and during the jump was allowed. Jump distance in centimeters between the starting line and heel of the posterior foot was recorded to the nearest one centimeter using a measuring tape. The participants had two test trials of which the best trial in terms of the longest jump distance was taken for further analysis. The standing long jump test showed high test–retest reliability for children aged 6–12 years with an ICC of 0.94^29^.

#### Upper limbs muscle power (PowerUP)

Power up was evaluated with the ball-push test. From a standing position, the participating children had to push a 1 kg medicine ball that was held tight right in front of the chest. The participants had to push the ball at maximal effort with both hands. The pushing distance in meters was recorded to the nearest ten centimeters using a measuring tape. The participants had two test trials of which the best test trial in terms of the longest pushing distance was taken for further analysis. The ball-push test was reliable (test–retest) for children aged 8–10 years with an ICC of 0.81^28^.

### Statistics

Pre- and post-processing of data were carried out in the R environment of statistical computing^30^ using the *tidyverse* package^31^. For statistical inference we relied on Linear Mixed Model analyses (LMM) with the *MixedModels* package^32^ in the *Julia* programming language (v 1.7.1)^33^.

**Figure 1:**
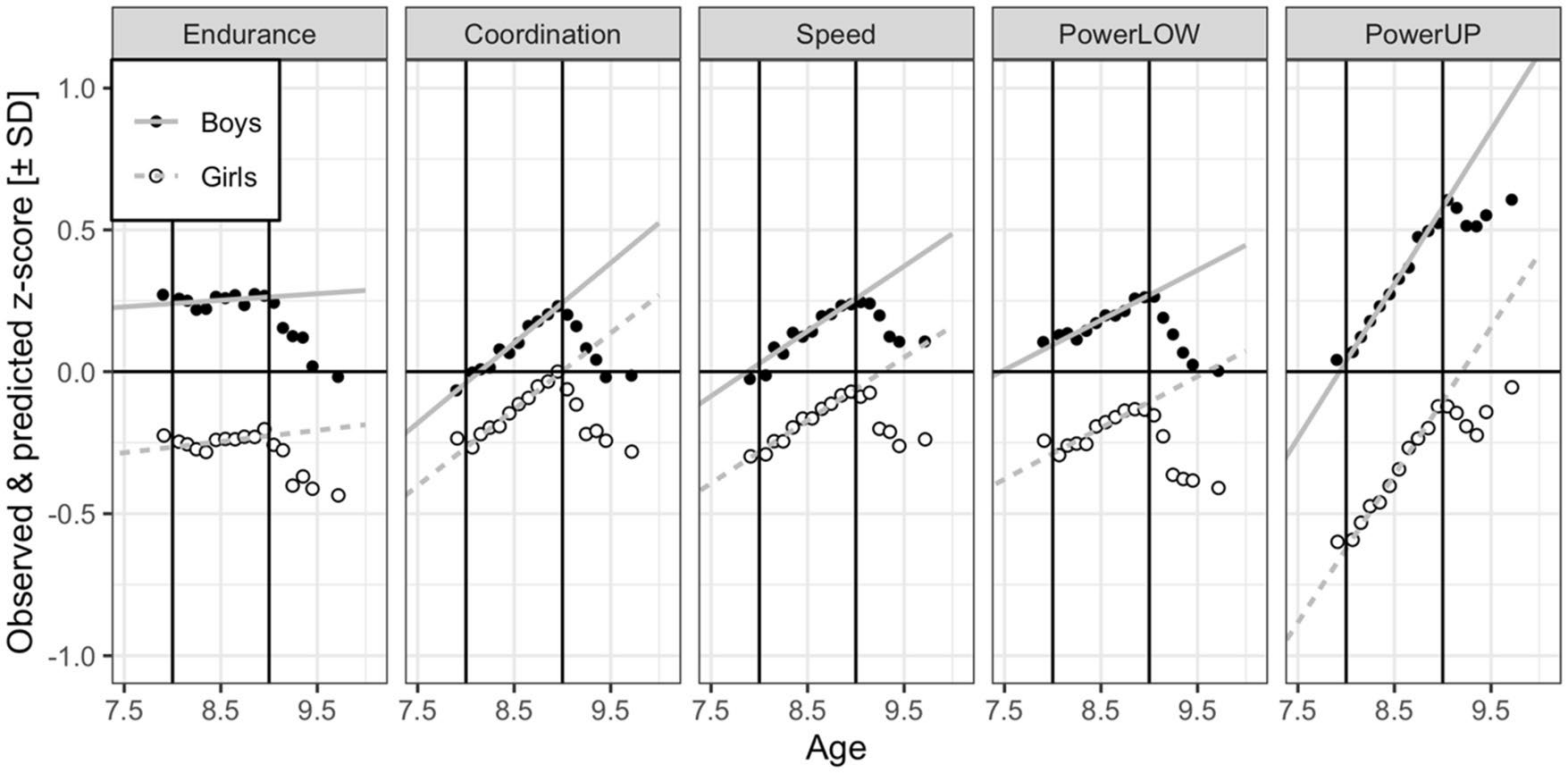
Observed z-scores for physical fitness development for boys (closed circles) and girls (open circles) aged 7.00–10.0 years. The lines represent the predicted z-scores for physical fitness development for boys (grey line) and girls (dashed grey line). Data were z-transformed. Endurance = cardiorespiratory endurance (i.e., 6-min-run test), Coordination = star-run test, Speed = 20-m linear sprint test, PowerLOW = lower limbs muscle power (i.e., standing long jump test), PowerUP = upper limbs muscle power (i.e., ball-push test). Note that *delta* z-scores for younger-than-keyage boys and girls were aggregated over 7.00–7.99 years and that *delta* z-scores for older-than-keyage boys and girls were aggregated over 9.50–9.99 years. Points are binned observed child means. Coordination and speed times were converted from seconds to meters/seconds (i.e., pace scores; star-run test = 50.912 [m]/time [s]; 20-m linear sprint test = 20 [m]/time [s]). These transformations have the advantage that a large value is indicative of better physical fitness and that they remove skew in the distributions.

**Figure 2:**
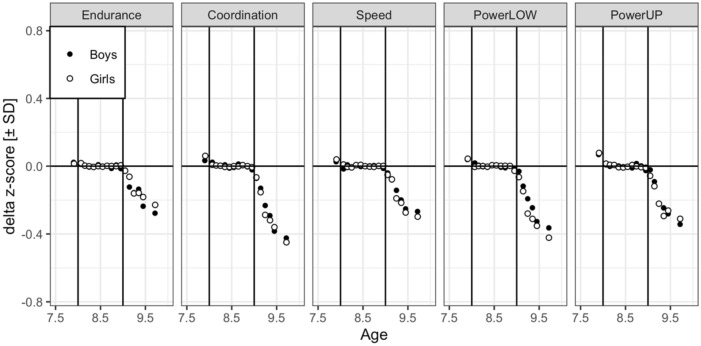
*Delta* z-score between observed and predicted physical fitness development for boys (closed circles) and girls (open circles) aged 7.00–10.0 years. Data were z-transformed. Endurance = cardiorespiratory endurance (i.e., 6-min-run test), Coordination = star-run test, Speed = 20-m linear sprint test, PowerLOW = lower limbs muscle power (i.e., standing long jump test), PowerUP = upper limbs muscle power (i.e., ball-push test). Note that *delta* z-scores for younger-than-keyage boys and girls were aggregated over 7.00–7.99 years and that *delta* z-scores for older-than-keyage boys and girls were aggregated over 9.50–9.99 years. Points are binned *delta* child means. Coordination and speed times were converted from seconds to meters/seconds (i.e., pace scores; star-run test = 50.912 [m]/time [s]; 20-m linear sprint test = 20 [m]/time [s]). These transformations have the advantage that a large value is indicative of better physical fitness and that they remove skew in the distributions.

For measures of cardiorespiratory endurance (i.e., 6 min run test), powerLOW (i.e., standing long jump test) and powerUP (i.e., ball push test), higher scores indicate better physical fitness. For measures of coordination (i.e., star run test) and speed (i.e., 20-m linear sprint test), a Box-Cox distributional analyses indicated that a reciprocal transformation brought scores in line with the assumption of a normal distribution^34^. Therefore, we converted scores from seconds to meters/seconds (i.e., pace scores; star run test = 50.912 [m]/time [s]; 20-m linear sprint test = 20 [m]/time [s]). These transformations had the advantage that a large value was indicative of good physical fitness for all five tests. Finally, z-scores were computed in two stages. In the first stage, we calculated z-scores within the test (i.e., 6-min-run test, star-run test, 20-m linear sprint test, standing long jump test, ball-push test) × sex (male, female) × group (YTK, OTK) cells and removed observations exceeding + /− 3 SDs (i.e., outliers). This is in accordance with a previous publication from the same research group^20^. In the second stage, we used means and SDs of the five fitness tests for keyage children from a previous study^20^ and computed the respective z-scores that were included in Figs. 1 and 2.

To compare YTK and OTK children`s development of physical fitness with that of keyage children, we predicted the physical fitness performance for ages 7 years and 0 months to 7 years and 11 months and 9 years and 0 months to 9 years and 11 months using LMM parameter estimates of the 108,295 keyage children (i.e., grey lines in Fig. 1), reported in Fühner et al^20^. Through this predication analyses we received the information about physical fitness performance of keyage children at the ages 7 years and 0 months to 7 years and 11 months and 9 years and 0 months and 9 years and 11 months. The model parameters comprised fixed effects for age, tests, sex, and their interactions, variance components (VCs) and correlation parameters (CPs) for GM and four test contrasts for the random factor child, VCs and CPs for GM, four test contrasts, sex, and age for the random factor school, and VCs for test and, age for the random factor assessment year. Details about model specification for these predictions are provided in Fühner et al.^20^ and in script: fggk22_lmm_pred.jl in the repository.

Through physical fitness testing in EMOTIKON, we obtained the actual physical fitness status of YTK children aged 7 years and 0 months to 7 years and 11 months and OTK children aged 9 years and 0 months to 9 years and 11 months. Please note that the classification of children into YTK, keyage, or OTK groups is based solely on children’s birthdate whereas children’s age is the difference between the date of test and their birthdate. Therefore, some YTK children were slightly older than 8 years and some OTK children were slightly younger than 9 years at the time of testing. Results did not change if non-keyage children aged between 8 and 9 years were excluded.

The difference between observed (i.e., obtained through physical fitness testing) and predicted z-scores (i.e., predicted data from keyage children [grey lines in Fig. 1]) yielded a *delta z-score* that was used as dependent variable in the following LMMs to compare physical fitness development of YTK and OTK children (i.e., obtained scores through physical fitness testing) with that of keyage children (i.e., predicted data).

We analyzed the data with separate LMMs for OTK and YTK children. The fixed effects included in the starting LMM were similar to the one reported by Fühner et al.^20^. Specifically, there were four sequential-difference fixed-effect contrasts for the five tests: (H1) coordination versus cardiorespiratory endurance, (H2) speed versus coordination, (H3) powerLOW versus speed, and (H4) powerUP versus powerLOW. We additionally included the effect of age (centered at 8 years and 6 months) as a second-order polynomial trend, the effect of sex (boys–girls), and all interactions between contrasts, age, and sex. We used a two-sided z-value > 2.0 as significance criterion for the interpretation of fixed effects.

The random effect structure included VCs and CPs of the *delta z-scores* for the five tests related to grouping (random) factors of child, school, and assessment year. Tests varied within children, schools, and assessment years; age and sex varied between children, but within schools and within assessment years. Therefore, in principle, VCs and CPs also include effects of age and sex for the factors school and assessment year.

#### LMM for older-than-keyage (OTK) children

The initial LMM included child (N = 26,540), school (N = 513), and assessment year (N = 9) as three random factors; the total number of observations (i.e., max = 5 per child) was 128,198. With three random factors, there was a need for selecting a random-effect structure that included theoretically relevant and reliable VCs and CPs but was also still supported by the data (i.e., was not overparameterized).

Parsimonious model selection occurred in two major steps without knowledge or consideration of fixed-effect estimates^35^; details are provided in script: fggk22_lmm_otk.jl in the repository. The random-effect structure of the parsimonious LMM of *delta* z-scores was expected to be simpler than the one for the LMM of Fühner et al.^20^ because the much smaller number of children and, importantly, because most of the school- and assessment-year-related random effects as well as fixed effect of age and sex were included in the predicted z-scores. We started with a model estimating VCs and CPs between *delta z-scores* of the five tests for children and VCs of *delta z-scores* for the five tests, age, and sex for school, and only varying intercept (GM) for assessment year. This LMM was well supported by the data. Increasing the complexity of the random-effect structure by adding CPs for school or adding VCs for assessment year did not improve the goodness of fit. Moreover, the school-related VC for sex and high-order fixed-effect interactions between test, age, and sex could be removed without loss of goodness of fit. As in Fühner et al.^20^, we also estimated the final model with an alternative post-hoc LMM parameterization to test main fixed effects of sex and age separately for each fitness test (i.e., we specified sex and age as nested within the five levels of the factor test).

#### LMM for younger-than-keyage (YTK) children

The LMM included child (N = 2586), school (N = 437), and assessment year (N = 9) as three random factors; the total number of observations (i.e., max = 5 per child) was 12,590. In the model selection process, we followed the model of OTK described above.

Parsimonious model selection occurred without knowledge or consideration of fixed-effect estimates^35^; details are provided in script: fggk22_lmm_ytk.jl in the repository. First, we applied the LMM of OTK to the data of YTK. This model was not supported by the data (i.e., overparameterized) because of the relatively small sample size of YTK (N = 2586) compared to OTK (N = 26,540). Indeed, the data supported only a LMM with a strongly reduced complexity, comprising (a) fixed effects on *delta z-scores* for the four contrasts of test, (b) VCs for the five *delta z-scores* for school and child, and (c) CPs for the five *delta z-scores* of child. Thus, there was no statistical support for fixed or random effects of age and sex for YTK children relating to *delta z-scores*.

## Results

Table 1 summarizes descriptive statistics for the three subsamples of third-graders. Statistics about keyage children refer to the sample reported in Fühner et al.^20^. Statistics about YTK and OTK children refer to the samples of this study.

**Table 1:** Descriptive statistics for younger-than-keyage, keyage, older-than-keyage children.

Sample	Physical fitness component	Sex	N schools	N child	Mean age [years]	SD age [years]	Mean score	SD score	Mean *delta*	SD *delta*
Keyage	Endurance [m]	Boys	513	51,116	8.56	0.28	1041.38	154.03	0	0.4
Keyage	Endurance [m]	Girls	511	52,821	8.55	0.28	967.72	132.50	0	0.3
Keyage	Coordination [m/s]	Boys	512	51,023	8.56	0.28	2.08	0.30	0	0.4
Keyage	Coordination [m/s]	Girls	510	52,886	8.55	0.28	2.01	0.27	0	0.3
Keyage	Speed [m/s]	Boys	513	51,700	8.56	0.28	4.58	0.42	0	0.4
Keyage	Speed [m/s]	Girls	512	53,259	8.55	0.28	4.45	0.39	0	0.4
Keyage	PowerLOW [cm]	Boys	513	52,141	8.56	0.28	129.41	19.53	0	0.4
Keyage	PowerLOW [cm]	Girls	509	53,856	8.55	0.28	122.00	18.44	0	0.4
Keyage	PowerUP [m]	Boys	514	52,254	8.56	0.28	3.99	0.70	0	0.4
Keyage	PowerUP [m]	Girls	512	54,070	8.55	0.28	3.50	0.63	0	0.3
OTK	Endurance [m]	Boys	511	14,870	9.35	0.25	1017.86	166.33	− 0.18	1.1
OTK	Endurance [m]	Girls	499	10,519	9.35	0.26	950.97	140.53	− 0.14	1.0
OTK	Coordination [m/s]	Boys	509	14,808	9.35	0.25	2.06	0.31	− 0.28	1.1
OTK	Coordination [m/s]	Girls	502	10,542	9.35	0.26	1.99	0.29	− 0.30	1.1
OTK	Speed [m/s]	Boys	511	15,010	9.36	0.25	4.58	0.44	− 0.17	1.1
OTK	Speed [m/s]	Girls	503	10,644	9.35	0.26	4.44	0.41	− 0.19	1.1
OTK	PowerLOW [cm]	Boys	511	15,137	9.35	0.26	127.83	21.08	− 0.23	1.2
OTK	PowerLOW [cm]	Girls	502	10,699	9.35	0.26	119.42	19.45	− 0.29	1.1
OTK	PowerUP [m]	Boys	511	15,236	9.36	0.25	4.13	0.75	− 0.22	1.1
OTK	PowerUP [m]	Girls	503	10,733	9.35	0.26	3.62	0.67	− 0.23	1.0
YTK	Endurance [m]	Boys	350	1087	7.85	0.19	1042.92	149.54	0.036	1.0
YTK	Endurance [m]	Girls	384	1408	7.88	0.18	973.11	132.88	0.035	1.0
YTK	Coordination [m/s]	Boys	350	1091	7.85	0.19	2.05	0.29	0.10	1.1
YTK	Coordination [m/s]	Girls	382	1397	7.87	0.18	1.99	0.26	0.10	1.0
YTK	Speed [m/s]	Boys	349	1097	7.85	0.19	4.51	0.40	0.035	1.1
YTK	Speed [m/s]	Girls	385	1423	7.87	0.18	4.42	0.39	0.070	1.0
YTK	PowerLOW [cm]	Boys	350	1112	7.85	0.19	128.54	18.48	0.082	1.1
YTK	PowerLOW [cm]	Girls	384	1433	7.87	0.18	121.77	18.06	0.078	1.0
YTK	PowerUP [m]	Boys	348	1111	7.85	0.19	3.79	0.70	0.12	1.0
YTK	PowerUP [m]	Girls	384	1431	7.88	0.18	3.33	0.61	0.14	0.9

N = sample size, SD = standard deviation, *delta* = difference between observed (i.e., obtained through physical fitness testing) and predicted z-scores (i.e., predicted data from keyage children [grey lines in Fig. 1]), Endurance = cardiorespiratory endurance (i.e., 6-min-run test), Coordination = star-run test, Speed = 20-m linear sprint test, PowerLOW = lower limbs muscle power (i.e., standing long jump test), PowerUP = upper limbs muscle power (i.e., ball-push test), OTK = older-than-keyage children (i.e., aggregated over 9 years and 0 months to 9 years and 11 months), YTK = younger-than-keyage children (i.e., aggregated over 7 years and 0 months to 7 years and 11 months). Coordination and speed times were converted from seconds to meters/seconds (i.e., pace scores; star-run test = 50.912 [m]/time [s]; 20-m linear sprint test = 20 [m]/time [s]). These transformations have the advantage that a large value is indicative of better physical fitness.

Figure 1 displays the observed (points) and predicted (lines) physical fitness development for YTK boys and girls aged 7 years and 0 months to 7 years and 11 months and OTK boys and girls aged 9 years and 0 months to 9 years and 11 months. The predicted z-scores for keyage children aged 8 years and 0 months to 8 years and 11 months are located on the predicted lines. There is a slight overlap between groups at 8- and 9-year boundaries due to birthdate determining the classification of children into keyage groups and age being measured as the difference between age at test and birthdate.

Figure 2 displays the *delta z-scores* between observed and predicted physical fitness development for YTK boys and girls aged 7 years and 0 months to 7 years and 11 months and OTK boys and girls aged 9 years and 0 months to 9 years and 11 months. The *delta z-scores* for keyage children aged 8 years and 0 months to 8 years and 11 months are represented in the horizontal zero line. The z-scores for OTK and YTK children will be described in the next sections.

### Physical fitness of older-than-keyage children (OTK)

Table 2 displays statistics for fixed effects of age (linear and quadratic) and sex as well as their interactions with the four test contrasts for LMM of OTK children.

**Table 2:** Fixed-effect estimates of linear mixed model for older-than-keyage (OTK) children.

Source of variance	Fixed-effect estimates	Standard error	z-values
**Main effects**			
Grand mean (intercept)	0.348	0.068	5.08*
H1: coordination versus endurance	0.230	0.091	2.51*
H2: speed versus coordination	− 0.025	0.083	− 0.30
H3: powerLOW versus speed	0.029	0.075	0.39
H4: powerUP versus powerLOW	− 0.022	0.094	− 0.23
Age (linear)	− 1.014	0.152	− 6.68*
Age (quadratic)	0.357	0.079	4.50*
Sex	0.015	0.011	1.43
**Age (linear) × Fitness component**			
H1: coordination versus endurance	− 0.693	0.205	− 3.38*
H2: speed versus coordination26	0.180	0.187	0.96
H3: powerLOW versus speed	− 0.181	0.168	− 1.08
H4: powerUP versus powerLOW	0.089	0.211	0.42
**Age (quadratic) × Fitness component**			
H1: coordination versus endurance	0.294	0.108	2.73*
H2: speed versus coordination	− 0.034	0.098	− 0.34
H3: powerLOW versus speed	0.059	0.088	0.67
H4: powerUP versus powerLOW	− 0.014	0.111	− 0.13
**Sex × Fitness component**			
H1: coordination versus endurance	0.052	0.015	3.41*
H2: speed versus coordination	− 0.001	0.014	− 0.10
H3: powerLOW versus speed	0.042	0.012	3.38*
H4: powerUP versus powerLOW	− 0.044	0.016	− 2.80*

H1–H4 = hypothesis 1–4, endurance = cardiorespiratory endurance (i.e., 6 min run test), coordination = star run test, speed = 20-m linear sprint test, powerLOW = lower limbs muscle power (i.e., standing long jump test), powerUP = upper limbs muscle power (i.e., ball push test), * = z-value > 2.0, linear mixed model random factors: assessment years (9), schools (513), children (26,540), observations = 128,198 (missing = 3.4%). For estimates of variance components and correlation parameters see Table 3.

The overall negative linear trend for age (z = − 6.68) and positive quadratic trend of age (z = 4.50) were significant. The positive quadratic trend of age indicates that the difference between predicted and observed physical fitness becomes more negative initially, but plateaus with even a slight reduction of *delta z-scores* for the oldest children (see Fig. 2).

Furthermore, the main effect of contrast H1 was significant (z = 2.51) indicating that the main effect was larger for coordination than for cardiorespiratory endurance. The LMM tested the interactions of linear and quadratic age with the four test contrasts, that is whether slopes in neighboring panels in Fig. 2 (averaged across sex) were parallel. The slope can be equated with the developmental rate. Indeed, one of four interaction was significant (see second and third block of Table 2) the linear age developmental rate was larger for cardiorespiratory endurance than coordination (H1; z = − 3.38) and the quadratic age developmental rate was larger for coordination than cardiorespiratory endurance (H1; z = 2.73).

Three of the test contrasts interacted with sex. First, the *delta z-score* was more negative for boys than girls for cardiorespiratory endurance and more negative for girls than boys for coordination (z = 3.41, see Table 1). The post-hoc LMM revealed significantly less severe *delta z-scores* for girls (− 0.14) than boys (− 0.18) for cardiorespiratory endurance (z =− 2.30). There was no significant sex difference for the *delta z-score* for coordination (z = 1.38). Second, the negative difference between boys and girls in the *delta z-score* was larger for powerLOW than speed (z = 3.38). The post-hoc LMM revealed a significant sex difference (favoring boys) only for powerLOW (z = 3.90; boys: − 0.23, girls: − 0.29; see Table 1). There was no significant sex difference for speed (z = 1.19). Third, the same powerLOW sex difference was the source of the significant interaction for the fourth contrast (z = − 2.80). There was no significant sex difference for powerUP (z = 1.12).

Table 3 lists estimates of VCs for children and for school. The delta z-scores VCs were large for children (0.88–0.94) and small for schools (0.06–0.09).

**Table 3:** Variance components for older-than-keyage (OTK) children.

Fitness component	GM	Endurance	Coordination	Speed	PowerLOW	PowerUP	Age
Assessment year	0.03						
School		0.08	0.07	0.07	0.06	0.07	0.09
Child (OTK)		0.92	0.94	0.94	0.98	0.88	

Endurance = cardiorespiratory endurance (i.e., 6 min run test), Coordination = star run test, Speed = 20-m linear sprint test, PowerLOW = lower limbs muscle power (i.e., standing long jump test), PowerUP = upper limbs muscle power (i.e., ball push test), OTK = older-than-keyage children. VC for Residual = 0.65.

### Physical fitness of younger than keyage (YTK) children

Table 4 displays estimates and test statistics for fixed effects of the four test contrasts. Figure 2 displays the *delta z-scores* between observed and predicted physical fitness development for YTK boys and girls aggregated over 7 years and 0 months to 7 years and 11 months.

**Table 4:** Fixed-effect estimates of the linear mixed model for younger-than-keyage (YTK) children.

Source of variance	Fixed-effect estimates	Standard error	z-values
**Main effects**			
Grand mean (intercept)	0.082	0.016	5.09*
H1: coordination versus endurance	0.074	0.024	3.05*
H2: speed versus coordination	− 0.055	0.022	− 2.49*
H3: powerLOW versus speed	0.026	0.021	1.27
H4: powerUP versus powerLOW	0.060	0.024	2.47*

H1–H4 = hypothesis 1–4, endurance = cardiorespiratory endurance (i.e., 6 min run test), coordination = star run test, speed = 20-m linear sprint test, powerLOW = lower limbs muscle power (i.e., standing long jump test), powerUP = upper limbs muscle power (i.e., ball push test), * = z-value > 2.0, linear mixed model random factors: schools (437), children (2586), observations = 12,590 (missing = 2.6%). For estimates of variance components and correlation parameters see Table 5.

The grand mean was significant (z = 5.09). Furthermore, three of the four main effects of contrasts were significant: the main effect was larger for coordination than cardiorespiratory endurance (H1; z = 3.05), larger for coordination than speed (H2; z = − 2.49) and larger for powerUP than powerLOW (H4; z = 2.47), which can also be seen in Fig. 2.

Table 5 lists estimates of VCs between *delta z-scores* for children and for school. The *delta z-scores* VCs were large for children (0.83–0.89) and small for schools (0.09–0.12).

**Table 5:** Variance components for younger-than-keyage (YTK) children.

Fitness component	Endurance	Coordination	Speed	PowerLOW	PowerUP
School	0.12	0.10	0.09	0.10	0.10
Child (YTK)	0.83	0.88	0.89	0.89	0.79

Endurance = cardiorespiratory endurance (i.e., 6 min run test), Coordination = star run test, Speed = 20-m linear sprint test, PowerLOW = lower limbs muscle power (i.e., standing long jump test), PowerUP = upper limbs muscle power (i.e., ball push test), YTK = younger-than-keyage (YTK) children. VC for Residual = 0.62.

## Discussion

The aim of this cross-sectional study was to examine physical fitness of YTK and OTK children versus keyage children in a representative sample of German primary school children. Our findings indicate that (1) OTK children showed poorer performance compared to keyage children, especially for coordination, (2) OTK girls outperformed OTK boys, and (3) YTK children showed better results than keyage children, especially for coordination.

Several studies confirmed a linear increase in physical fitness performance with chronological age^9–11,15^. For instance, in a study with 424,328 Greek children and adolescents aged 6–18 years, Tambalis et al.^15^ reported a linear increase in physical fitness performance with age for cardiorespiratory endurance (i.e., 20-m shuttle run test), lower limbs muscle power (i.e., standing long jump test), flexibility (i.e., sit-and-reach test), muscular strength (i.e., sit-ups test), and agility (i.e., 10 × 5 m shuttle run test). The development of physical fitness of keyage children (see predicted gray lines in Fig. 1) is in accordance with the above reported results. For keyage children, physical fitness performance increased linearly with age. However, the development of physical fitness for OTK children is different. Poor performance was found in OTK children aged 9 years and 0 months to 9 years and 11 months compared with age-matched keyage children for all components of physical fitness, especially for coordination. This could be due to the fact that third graders aged 9 years and 0 months to 9 years and 11 months (i.e., OTK children) are not representative for the “average” age-matched keyage child which is why we observed a deviation from the typically reported fitness development with age in this cohort^9–11,15^. We do not know the exact circumstances which lead to the delayed enrollment into first grade or to the repetition of a school year. According to our results, we can only speculate that maybe a delay in cognitive development might be the reason why children are late enrolled into first grade or must repeat a school class. These results are in line with a study of Urschitz et al.^23^ who examined differences in academic performance. These authors observed that poor academic performance significantly increased with age for mathematics, science, reading, spelling, and handwriting in a sample of 1144 German third graders. Of note, children who repeated a school class were more prone to poor academic performance. These results were confirmed by other studies for academic performance^21,22^. Interestingly, in our study OTK girls showed better performance compared to OTK boys which is in accordance with Urschitz et al.^23^. These authors reported that except for mathematics, boys showed a larger prevalence for poor academic performance compared with girls^23^. As girls mature approximately two years earlier than boys, the better performance of girls compared to boys might be influenced by biological maturation. Girls enter the adolescent growth spurt at approximately ten years of age and peak height velocity at 12 years, whereas boys enter the growth spurt on average at age 12 and peak height velocity at 14^36^.

In contrast, YTK children outperformed keyage children especially in tests requiring motor coordination. Again, we do not know the exact circumstances which resulted in early enrollment into first grade or reasons for skipping a school year. According to our results, we speculate that accelerated cognitive development could be a reason why early enrolled children skip a school year. This is supported by the fact that in this study, YTK children showed the best performance in the coordination test which has an inherent large cognitive demand. Moreover, findings from Martin^22^ point in a similar direction by showing that in a cohort of 3684 Australian high school students, YTK children outperformed keyage children in academic performance.

Our study is not without limitations. First, anthropometric factors such as body mass, body height, and sitting height were not assessed in this study so that associations between anthropometric factors, biological maturation, and physical fitness could not be calculated. These factors would have provided additional insight as there is strong evidence that children’s physical fitness is associated with anthropometric characteristics^37–39^ and biological maturation^36^. One explanation of the deviation of YKT and OKT children might be a difference between chronological and biological age. It appears plausible to argue that YKT children may be more mature and that OKT children are biologically somewhat younger than indicated by their chronological age. Thus, in a hypothetical plot of performance over biological age, the linear trend may well hold for all children. Second, we predicted the performance of the YTK and OTK children based on a linear extrapolation recently reported by Fühner et al.^20^. However, we do not know if this linear extrapolation exactly fits to the data of keyage children aged 7 years and 0 months to 7 years and 11 months/9 years and 0 months to 9 years and 11 months as we do not have such longitudinal data. Third, we cannot parse out the exact number of OTK children that were late enrolled or repeated a school class.

To sum up, this study is the first study that examined differences in physical fitness development of YTK and OTK children compared to keyage children. Our study findings complement results reported in the literature on the development of academic performance in youth^21–23^. Politicians and decision makers, schools, (physical education) teachers, and parents should be aware that OTK versus keyage children showed poorer physical fitness performance. This is a novel and somehow unexpected result. Therefore, OTK children should be specifically promoted through additional health and fitness programs to compensate their deficits in physical fitness to enable a holistic development. Furthermore, the assessment of physical fitness should be performed regularly to tailor the contents of physical education classes based on the results of physical fitness assessments (e.g., data driven physical education classes). More specifically, the physical fitness status of OTK children should be monitored regularly over time to evaluate whether e.g., additional health and fitness programs already helped to compensate the observed deficits in physical fitness.

Given that reference values for the grading of physical fitness is only available for keyage children, raw data from this study can be used to calculate age-, sex-, and timing of school enrollment-specific percentile values. The respective data should be useful for (physical education) teachers or researchers to individually evaluate and grade children´s physical fitness development.

The EMOTIKON test battery is easy-to-administer, cost effective, and it requires only minimal equipment that is usually available in gyms (e.g., stopwatch, measuring tape, medicine ball, pylons). Therefore, physical education teachers, coaches, or researchers can use the EMOTIKON test battery to evaluate children’s physical fitness and use the results to promote health- and skill-related physical fitness during physical education.

## Data Availability

The datasets generated and analyzed during the current study as well as Julia and R scripts are available in the Open Science Framework (OSF) repository: https://osf.io/dmu68/?view_only=240bdab8f1be4d8384acf9356ee50f8b.
